# A quantum Samaritan’s dilemma cellular automaton

**DOI:** 10.1098/rsos.160669

**Published:** 2017-06-14

**Authors:** Ramón Alonso-Sanz, Haozhen Situ

**Affiliations:** 1 Technical University of Madrid, ETSIAAB (Estadistica, GSC), C. Universitaria, Madrid 28040, Spain; 2 College of Mathematics and Informatics, South China Agricultural University, Guangzhou 510642, People’s Republic of China

**Keywords:** quantum games, noise, Samaritan dilemma, cellular automata

## Abstract

The dynamics of a spatial quantum formulation of the iterated Samaritan’s dilemma game with variable entangling is studied in this work. The game is played in the cellular automata manner, i.e. with local and synchronous interaction. The game is assessed in fair and unfair contests, in noiseless scenarios and with disrupting quantum noise.

## 1. The classic and quantum Samaritan’s dilemma

**Figure 1.**
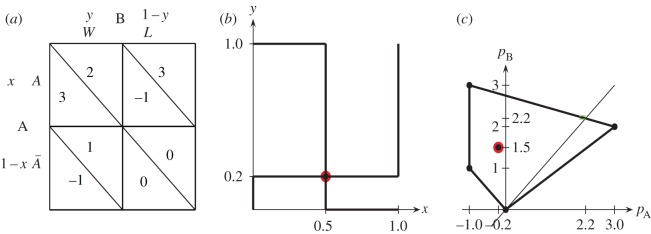
Pay-off matrices (*a*), reaction functions (*b*) and pay-off region in the Samaritan’s dilemma (*c*) studied in this article.

The Samaritan’s dilemma (SD) is a non-zero sum, asymmetric game played by two players: the charity player A and the beneficiary player B. Player A may choose Aid/No Aid, whereas player B may choose Work/Loaf. The Samaritan’s dilemma arises in the act of charity. The charity wants to help (Aid) people in need. However, the beneficiary may simply rely on the handout (Loaf) rather than try to improve their situation (Work). This is not anticipated by the charity. Many people may have experienced this dilemma when confronted with people in need. Although there is a desire to help them, there is the recognition that a handout may be harmful to the long-run interests of the recipient [1–5]. Following Huang *et al.* [6], Ozdemir *et al.* [7] and Rasmussen [8], we adopt here the pay-off matrices **P**_A_ and **P**_B_ given in figure 1*a*.

### 1.1. The classic context

In conventional classic games, both players decide independently their probabilistic strategies **x**=(*x*,1−*x*)^′^ and **y**=(*y*,1−*y*)^′^. As a result, the expected pay-offs (*p*) in the SD game are

1.1 $$\begin{array}{rl} p_{A}(x,y) & ={\text{x}}^{\prime }{\text{P}}_{A}\text{y}=(5y-1)x-y\;\mathrm{and}\\ p_{B}(x,y) & ={\text{x}}^{\prime }{\text{P}}_{B}\text{y}=(1-2x)y+3x. \end{array}$$

The SD belongs to the class of the so-called discoordination games, i.e. games with no pair of pure strategies in Nash equilibrium (NE), where the one player’s incentive is to coordinate (charity (*A*,*W*)), while the other player tries to avoid this (beneficiary (*A*,*L*)). Figure 1*b* shows the reaction functions whose intersection determines the NE, i.e. **x** is a best response to **y** and **y** is a best response to **x**. In our case, (*x*=0.5; *y*=0.2), with associated pay-offs *p*_A_=−0.2, *p*_B_=1.5. Figure 1*c* shows the pay-offs region of the studied game, which turns out negative for player A in points such as the NE, whereas this does not happen for player B.

Let us remark here then, that the pay-offs of the SD are biased towards the beneficiary player B. In other words, the dilemma of the Samaritan game (sometimes referred to as the Welfare game) is somehow only that of the charity (or Samaritan) player A.

In a different game scenario, that of correlated games, an *external* probability distribution $\Pi =(\begin{array}{cc} \pi_{11} & \pi_{12}\\ \pi_{21} & \pi_{22} \end{array})$ assigns probability to every combination of player choices [9]. Thus, the expected pay-offs in the SD are

1.2 $$\begin{array}{rl} p_{A} & =3\pi_{11}-\pi_{12}-\pi_{21}\\ \text{and}\;p_{B} & =2\pi_{11}+3\pi_{12}+\pi_{21}. \end{array}\}$$

The quantum game approach described in the next subsection, participates of both the independent players (1.1) and of the correlated games (1.2) models.

### 1.2. Quantum games

In the quantization scheme introduced by Eisert *et al.* [10] (EWL for short), the classical pure strategies are assigned two basic vectors |0〉 and |1〉, respectively, in a two-level Hilbert space. The state of the game is a vector in the tensor product space spanned by the basis vectors |00〉, |01〉, |10〉, |11〉. The seminal paper [10] deals with the Prisoner’s Dilemma, so that the classical pure strategies for both players are that of Cooperation and Defection, referred to as *C* and *D*, respectively. We will keep this strategy codification in this section, although in the context of the SD game *C* standing for Aid for the Samaritan—Work for the beneficiary, and *D* standing for no Aid for the Samaritan—Loaf for the beneficiary. The EWL quantum protocol, described below, includes the classical approach as a particular case, but with the purely classical strategies referred to as $\hat{D}$ and $\hat{C}=I=(\begin{array}{cc} 1 & 0\\ 1 & 0 \end{array})$, so that the *hat* operator mark indicates by itself that a quantum approach is taken into account.

The EWL protocol starts with an initial entangled state $\left|\psi_{i}\rangle =\hat{J}|00\right\rangle$, where the symmetric unitary operator $\hat{J}=\exp(i(\gamma /2){\hat{D}}^{⊗2}),$ $\hat{D}=(\begin{array}{cc} 0 & 1\\ -1 & 0 \end{array})$, *entangles* the player’s qubits. The *entanglement factor* *γ* varies in the [0,*π*/2] interval, tuning the degree of entanglement. The initial state then becomes: $\left|\psi_{i}\rangle =\cos(\gamma /2)|00\rangle +i\sin(\gamma /2)|11\right\rangle$. Consequently, the initial density matrix is

1.3 ρi=|ψi⟩⟨ψi|=( cos2⁡γ200−i cos⁡γ2sin⁡γ200000000i cos⁡γ2sin⁡γ200sin2⁡γ2).

The players perform independently their quantum strategies as local unitary operators (${\hat{U}}_{A}$,${\hat{U}}_{B}$) in the SU(2) space. With the only exception of §4, we will consider here the two-parameter subset (2P) of SU:

1.4 U^(θ,α)=(eiα cos(θ2)sin(θ2)−sin(θ2)e−iα cos(θ2)),θ∈[0,π]α∈[0,π2].

After the application of these strategies, the state of the game evolves to $\left|\psi_{f_{o}}\rangle =({\hat{U}}_{A}⊗{\hat{U}}_{B})\hat{J}|00\right\rangle$. Prior to measurement, the ${\hat{J}}^{†}$ gate is applied and the state of the game becomes: $\left|\psi_{f}\rangle ={\hat{J}}^{†}({\hat{U}}_{A}⊗{\hat{U}}_{B})\hat{J}|00\right\rangle$. This follows a pair of Stern–Gerlach type detectors for measurement. As a result, the elements of $\Pi =(\begin{array}{cc} \rho_{11} & \rho_{22}\\ \rho_{33} & \rho_{44} \end{array})$ are obtained as the diagonal elements of the final density matrix:

1.5 $$\rho_{f}=|\psi_{f}\rangle \langle \psi_{f}|={\hat{J}}^{†}({\hat{U}}_{A}⊗{\hat{U}}_{B})\rho_{i}{\left({\hat{U}}_{A}⊗{\hat{U}}_{B}\right)}^{†}\hat{J}.$$

**Figure 2.**
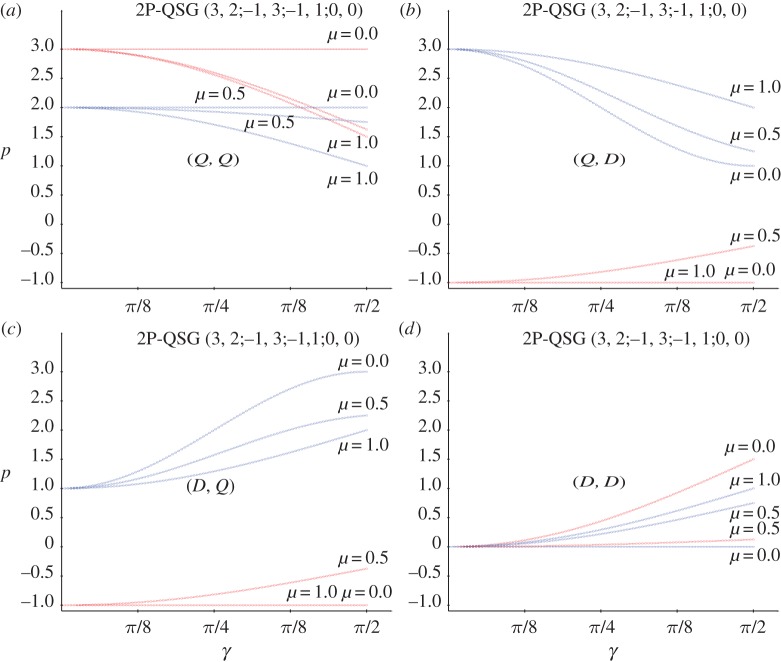
Pay-offs in a QSD with variable entanglement factor *γ*. (*a*) $\left(\hat{Q},\hat{Q}\right)$, (*b*) $\left(\hat{Q},\hat{D}\right)$, (*c*) $\left(\hat{D},\hat{Q}\right)$ and (*d*) $(\hat{D},\hat{D}).$ Three levels of noise (*μ*) are considered in each frame. Red: pay-offs of player A (Samaritan), blue: pay-offs of player B (beneficiary).

The outstanding strategy $\hat{Q}=\hat{U}(0,\pi /2)=(\begin{array}{cc} i & 0\\ 0 & -i \end{array})$ in a $\left\{\hat{Q},\hat{Q}\right\}$ contest gives rise to *π*_11_=1∀*γ*, and in consequence the pay-offs of the (*A*,*W*) choice. Thus, in the quantum Samaritan dilemma game (QSD) here studied, *p*_A_=3 and *p*_B_=2, as may be checked in the horizontal lines of figure 2*a* under the label *μ*=0.0.

The two-parameter (2P) model using the two-parameter operators given in (1.4) may be criticized as being just a subset of the general SU(2) space of unitary strategies which allows for three parameters. A subset that is not a closed set and fails to reflect any reasonable physical constraint [11,12]. As pointed out in the seminal paper dealing with the quantum Samaritan’s dilemma [7], the 2P-restriction in the original formulation of the EWL model is a subject of continuing discussions. But accordingly with that stated in [7], the 2P-EWL model is a good (and widely used) test-bed to show how the quantum approach in game theory may *solve* dilemmas, by allowing for NE strategies out of the scope of the classic approach [13]. Consequently, the structure of this study follows the way paved for the paper [14] (a sequel of the seminal paper [10]) dealing with the prisoner’s dilemma game, and more importantly regarding this study in [7]: one-parameter (classic, §1.1) → two-parameters (§§2 and 3) → three-parameters (§4).

### 1.3. Quantum noise

Real-world quantum information processing systems inevitably interact with the environment. The environmental noise may destroy the quantum properties and be harmful to our purpose of information processing. So it is important to take quantum noise into our consideration [15].

In the presence of noise, the shared state is corrupted before the players apply their strategies ${\hat{U}}_{A}⊗{\hat{U}}_{B}$. The noise effect on a single qubit can be described by CPTP (completely positive trace-preserving) maps: $N(\rho )=\sum_{i}K_{i}\rho K_{i}^{†}$, with $\sum_{i}K_{i}^{†}K_{i}=I$. We assume both qubits of the shared state are affected by the same kind of noise. As a result,

1.6 $$\rho^{*}=\sum_{i=1}^{2}\sum_{j=1}^{2}(K_{i}⊗K_{j})\rho_{f}{\left(K_{i}⊗K_{j}\right)}^{†}.$$

We will consider here the effect of amplitude-damping noise type:

1.7 $$K_{1}=\left(\begin{array}{cc} 1 & 0\\ 0 & \sqrt{1-\mu } \end{array}\right)\;\mathrm{and}\;K_{2}=\left(\begin{array}{cc} 0 & \sqrt{\mu }\\ 0 & 0 \end{array}\right),$$

where *μ*∈[0,1] represents the strength of noise.

The final density matrix for $\mu =\frac{1}{2}$ becomes,

$$\rho^{*}\left(\mu =\frac{1}{2}\right)=\left(\begin{array}{cccc} 1-\frac{3}{4}\sin^{2}\frac{\gamma }{2} & 0 & 0 & -i\frac{1}{4}\sin\gamma \\ 0 & \frac{1}{4}\sin^{2}\frac{\gamma }{2} & 0 & 0\\ 0 & 0 & \frac{1}{4}\sin^{2}\frac{\gamma }{2} & 0\\ i\frac{1}{4}\sin\gamma & 0 & 0 & \frac{1}{4}\sin^{2}\frac{\gamma }{2} \end{array}\right).$$

The pay-off dependence of *γ* in the SD of *Q* versus the pure strategies C and D with $\mu =\frac{1}{2}$ may be checked in figure 2 under the label $\mu =\frac{1}{2}$. In the (*Q*, *Q*) scenario in particular, the pay-offs become: $p_{A}^{QQ}=3-\frac{11}{4}\sin^{2}(\gamma /2)$ and $p_{B}^{QQ}=2-\frac{2}{4}\sin^{2}(\gamma /2)$, which equalize at $1=\frac{9}{4}\sin^{2}(\gamma /2)$, i.e. at $\gamma =2\arcsin\left(\sqrt{\frac{4}{9}}\right)=1.460$, giving *p*_A,B_=1.777. Beyond *γ*=1.460 it is $p_{A}^{QQ}<p_{B}^{QQ}$, as with full entangling where $p_{A}^{QQ}=\frac{13}{8}<p_{B}^{QQ}=\frac{14}{8}$.

With maximum noise it is

$$\rho^{*}(\mu =1)=\left(\begin{array}{cccc} 1 & 0 & 0 & 0\\ 0 & 0 & 0 & 0\\ 0 & 0 & 0 & 0\\ 0 & 0 & 0 & 0 \end{array}\right),$$

which leads to

$$\Pi^{QQ}=\left(\begin{array}{cc} \cos^{2}\frac{\gamma }{2} & 0\\ 0 & \sin^{2}\frac{\gamma }{2} \end{array}\right).$$

Permuting the columns, the rows and the rows and columns of *Π*^*QQ*^, generates *Π*^*QD*^, *Π*^*DQ*^ and *Π*^*DD*^, respectively. The pay-off dependences of *γ* in the SD of the pure strategies with full noise may be checked in figure 2 under the label *μ*=1. In particular, in the (*Q*,*Q*) context, pAQQ=3 cos2⁡(γ/2), pBQQ=2 cos2⁡(γ/2).

Let us remark that only amplitude-damping quantum noise will be under scrutiny in this study. The effect of other types of quantum noise [16] will be taken into account in future work. In particular that of phase-damping, structurally close to amplitude-damping as both damping noises have the same *K*_1_ Kraus operator, deferring in $K_{2}=(\begin{array}{cc} 0 & 0\\ 0 & \sqrt{\mu } \end{array})$ with phase damping. Mirroring the proximity of the two noise types, with full phase-damping noise, *Π*^*QQ*^ becomes diagonal as with amplitude damping, but with $\pi_{22}^{QQ}=\frac{1}{2}\sin^{2}\gamma$ instead of $\pi_{22}^{QQ}=\sin^{2}(\gamma /2)$. With *μ*=0.5 phase damping, *Π*^*QQ*^ also turns out diagonal ($\pi_{22}^{QQ}=\frac{1}{4}\sin^{2}\gamma$) so that *p*_A_>*p*_B_, ∀*γ*, at variance with what happens with amplitude damping where both pay-offs intersect as indicated before.

## 2. The spatialized quantum Samaritan dilemma game

In the spatial version of the quantum SD (QSD-CA), we deal with each player occupying a site (*i*,*j*) in a two-dimensional *N*×*N* lattice. In order to compare different types of players, two types of players, termed *A* and *B*, are to be considered. *A* and *B* players alternate in the site occupation in a chessboard form, so that every player is surrounded by four orthogonal adjacent partners (*A*−*B*, *B*−*A*), and four diagonal adjacent mates, i.e. players with the same role, either Samaritan or beneficiary (*A*−*A*, *B*−*B*). The game is played in the cellular automata (CA) manner, i.e. with uniform, local and synchronous interactions [17]. In this way, every player plays with his four adjacent partners, so that the pay-off $p_{i,j}^{\left(T\right)}$ of a given individual is the sum over these four interactions. The evolution is ruled by the (deterministic) imitation of the best paid neighbour, so that in the next generation, every generic player (*i*,*j*) will adopt the parameters of his mate player (*k*,*l*) with the highest pay-off among their mate neighbours.

All the simulations in this work are run up to *T*=200 in a *N*=200 lattice with periodic boundary conditions and five different initial random assignment of the (*θ*,*α*) parameter values in each scenario. In the subsequent figures, the results regarding the charity player A have been marked red, and those regarding the beneficiary player B have been marked blue. With the exception of figures 4, 9, and 16, the subsequent figures deal with the results obtained in QSD-CA simulations at *T*=200 with variable entanglement factor *γ* at three noise levels. These figures show in their left frame the mean pay-offs across the lattice ($\bar{p}$) of both player types, and in their right frame the mean parameter values across the lattice. The computations have been performed by a double precision Fortran code run on a mainframe.

Before presenting the results obtained regarding the Samaritan’s dilemma, let us point here that this study follows the way paved by previous studies dealing with CA simulations of prototypical games such as the symmetric Prisoner’s Dilemma (PD) [18–21] and the asymmetric Battle of the Sexes (BoS) [22–25]. Unlike the discoordination SD game, both the PD and the BoS have NE based on pure strategies, which marks a decisive qualitative difference in the study of the SD compared with those of the PD and the BoS.

**Figure 3.**
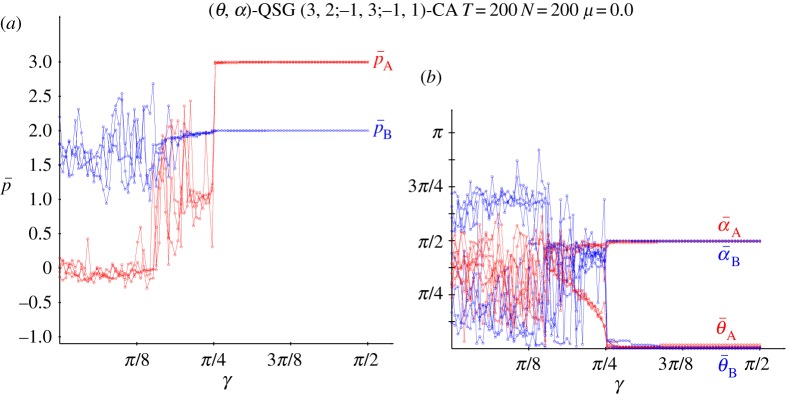
Five noiseless simulations at *T*=200 of a QSD-CA with variable entanglement *γ*. (*a*) Mean pay-offs and (*b*) mean quantum parameter values (*θ*,*α*) across the lattice.

Figure 3 deals with the results obtained in a QSD-CA simulation free of noise. The beneficiary player B clearly overrates the charity player A for low values of the entanglement, so that for *γ* up to just passed *π*/8, ${\bar{p}}_{A}$ oscillates slightly below zero, whereas ${\bar{p}}_{B}$ oscillates slightly over around 1.5, thus not far from (−0.2,1.5), the pay-offs in classic NE. If *γ*=0 (or if *α*_A_=*α*_B_=0), *Π* becomes factorizable as in the *classic* game with independent strategies (1.1), i.e. *Π*=**xy**^′^, with $x=\cos^{2}\theta_{A}/2$, $y=\cos^{2}\theta_{B}/2$, which makes the *α* parameters irrelevant. It is $\theta_{A}^{\mathrm{NE}}=2\arccos(0.5)=\pi /2=1.570$, $\theta_{B}^{\mathrm{NE}}=2\arccos(0.2)=2.214$, not far from 3*π*/4=2.35^1^ . Over *π*/8 and before *π*/4, figure 3*b* shows that ${\bar{\theta }}_{A}$ gradually tends to zero as *γ* increases, which is reflected in a stabilization of ${\bar{p}}_{A}$ in figure 3*a*. From *γ*=*π*/4, and before *π*/4, ${\bar{\theta }}_{A}={\bar{\theta }}_{B}=0$ and ${\bar{\alpha }}_{A}={\bar{\alpha }}_{B}=\pi /4$, i.e. both players adopt the $\hat{Q}$ strategy. Remarkably, the pair $\left\{\hat{Q},\hat{Q}\right\}$ is the only pair in NE for *γ*>*π*/4. This is so because, (i) *p*^*QQ*^_*B*_=2 and pBQD=3 cos2γ+sin2γ=3−2sin2γ, so that $p_{B}^{QQ}>p_{B}^{QD}$ for *γ*>γ*=*π*/4 and (ii) *p*^*QQ*^_*A*_=3 and $p_{A}^{DQ}=-1\sin^{2}\gamma -\cos^{2}\gamma =-1$, so that $p_{A}^{QQ}>p_{A}^{DQ}\;\forall \gamma$.

Thus, the main finding derived from figure 3 is that the imitation dynamics in CA enables the emergence of NE in the SD: the NE that there is in the classic game with low entanglement and that of the pair (*Q*,*Q*) with high entanglement. The latter reverses the SD structural trend in favour of the beneficiary player, inducing the highest possible sum of pay-offs. One may say that the *Q* strategy resolves the dilemma, though the sceptical reader might argue that as *Q* has no *real* component (*θ*=0), the dilemma is resolved in the *imaginary* … world.

**Figure 4.**
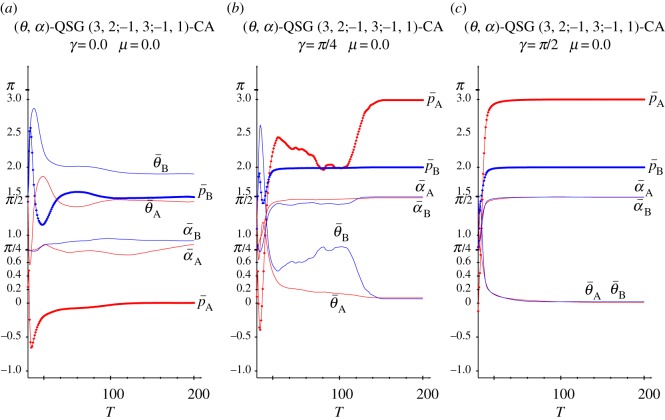
Dynamics in three simulations in the QSD-CA scenario of figure 3. (*a*) *γ*=0, (*b*) *γ*=*π*/4 and (*c*) *γ*=*π*/2.

Figure 4 shows the dynamics in simulations in the QSD-CA scenario of figure 3 with *γ*=0 (figure 4*a*), *γ*=*π*/4 (figure 4*b*) and *γ*=*π*/2 (figure 4*c*). As a result of the initial random assignment of the parameter values, it is initially in both frames: $\bar{\theta }≃\pi /2=1.570$, $\bar{\alpha }≃\pi /8=0.785$. With *γ*=0, after a short transition time, both the parameters and mean pay-offs stabilize their values fairly soon, the latter close to the pay-offs of NE (−0.2,1.5). In the *γ*=*π*/2 dynamics in figure 4*c*, both $\bar{\theta }$’s rocket to *π*, and both $\bar{\alpha }$’s plummet to zero, i.e. both players play the *Q* strategy and in consequence the mean pay-offs of A and B players stabilize at (3.0,2.0) in a straightforward manner. Remarkably, the parameter tendencies heavily emerge from the very beginning, despite the full range of parameters initially accessible in the CA local interactions. In figure 4*b*, with *γ*=*π*/4, the threshold for the emergence of *Q*, the parameter stabilization is achieved also in fast manner, with the exception of ${\bar{\theta }}_{A}$, whose trend towards zero is achieved beyond *T*=100 in a rather unexpected way which determines the sudden leap of ${\bar{p}}_{A}$ towards 3.0.

**Figure 5.**
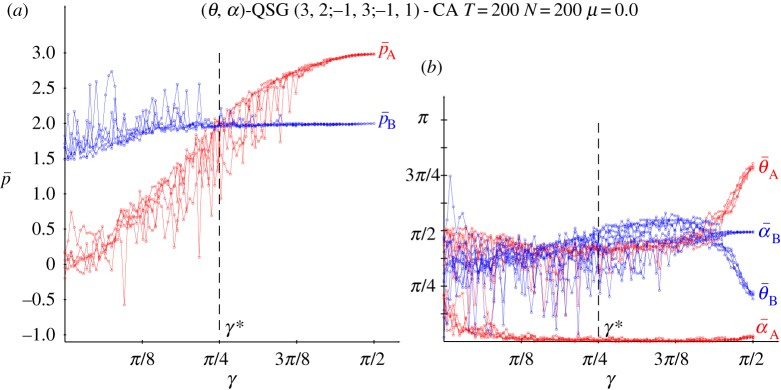
Five noiseless simulations at *T*=200 of a QSD-CA with $\left|\psi_{i}\rangle =\hat{J}|01\right\rangle$ and variable entanglement *γ*. (*a*) Mean pay-offs and (*b*) mean quantum parameters.

The seminal reference [7] analyses also a variant of the original EWL model that considers as initial state $\left|\psi_{i}\rangle =\hat{J}|01\right\rangle$ instead of $\left|\psi_{i}\rangle =\hat{J}|00\right\rangle$. Four pair of strategies in NE are reported in [7] in the full entangled model starting from |01〉. The four NE pairs induce the (3,2) pay-offs and have in common that: *α*_A_=0 and *α*_B_=*π*/2. Figure 5 shows that CA simulations tend to induce ${\bar{\alpha }}_{A}=0$ and ${\bar{\alpha }}_{B}=\pi /2$ for not low *γ* and select the NE pair with *θ*_A_=3*π*/4 and *θ*_B_=*π*/4 for high entanglement. The most distinctive difference of figure 5 compared with figure 3 is that of the fairly monotonic increase of ${\bar{p}}_{A}(\gamma )$ and the lower variation of the five values of ${\bar{p}}_{A}$ for low values of *γ*.

Let us point out here that non-factorizable ***Π*** may be generated from independent strategies **(x, y)** in the classic context, without resorting to any quantum approach. Thus, for example, an ad hoc method based in the external parameter 0≤*k*≤1 is given in [26], and shown as follows:

2.1 $$\begin{array}{rlrl} \pi_{11} & ={\left(2k-1\right)}^{2}xy & \;\pi_{12} & =(1-k)x(1-y)+k(1-x)y\\ \text{and}\;\pi_{21} & =(1-k)(1-x)y+kx(1-y) & \;\pi_{22} & =(1-x)(1-y)+4k(1-k)xy. \end{array}\}$$

**Figure 6.**
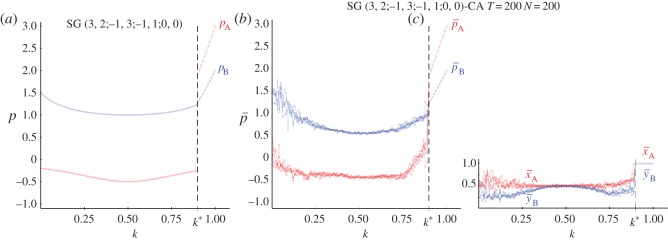
Simulations in the SDG with joint probabilities generated by the mechanism given in (2.1). (*a*) Pay-offs of both players with strategy pairs in NE, (*b*) mean pay-offs and mean values of *x* and *y* (*c*) in five simulations at *T*=200 of a SDG-CA.

With the joint probabilities generated as in (2.1), the pairs in NE in the SDG are given in (2.2), where the threshold k* emerges from the *x*≤1 restraint. The pay-offs of both players in the SDG with strategy pairs in NE under the model (2.1) are plotted in figure 6*a*, being, *p*_A_=−1/(2+3(2*k*−1)^2^), *p*_B_=((3−2*k*)(1+2*k*))/(4−2(2*k*−1)^2^) for *k*≤k*=0.89.

2.2 $$\text{NE}(k):\begin{array}{cc} \left(x=\frac{1+2k}{4-2{\left(2k-1\right)}^{2}},y=\frac{1}{2+3{\left(2k-1\right)}^{2}}\right) & k\leq k^{*}=0.89\\ (x=1,y=1)\;\;\;\;\;\; & k\geq k^{*}=0.89. \end{array}$$

Figure 6*b*,*c* shows the mean pay-offs (figure 6*b*) and mean values of *x* and *y* (figure 6*c*) in five simulations at *T*=200 of a SDG-CA. The form of the pay-offs curves in the CA simulations mimic remarkably well that achieved with strategies in NE in conventional (non-CA) simulations (figure 6*a*) for threshold *k*<k*. For *k*≥k* the CA simulations produce the exact values $\bar{x}=\bar{y}=1$, which induce *π*_11_=(2*k*−1)^2^, *π*_12_=*π*_21_=0, *π*_22_=4*k*(1−*k*) and consequently the pay-offs of the (A,W) pair, i.e. *p*_A_=3(2*k*−1)^2^, *p*_B_=2(2*k*−1)^2^, which show a fairly linear aspect for *k*>k*, reaching *p*_A_=3,*p*_B_=2 for *k*=1 (as achieved with *k*=0).

### 2.1. Quantum noise

**Figure 7.**
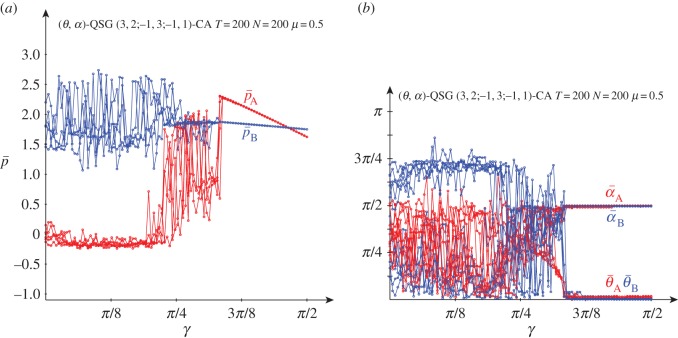
Five simulations at *T*=200 of a QSD-CA with variable entanglement *γ* and *μ*=0.5 noise factor. (*a*) Mean pay-offs and (*b*) mean quantum parameter values.

Figure 7 deals with the results obtained in a QSD-CA simulation with *μ*=0.5 noise. The general form of the graphs of $\left({\bar{p}}_{A},{\bar{p}}_{B}\right)$ for not high entanglement shown in the simulations free of noise (figure 3) remains in figure 7 up to nearly *γ*=*π*/4 with *μ*=0.5 noise. From approximately *γ*=*π*/4, ${\bar{\theta }}_{A}$ gradually tends to zero, so that, as in the noiseless simulation, the mean pay-off of the player A is notably stabilized. The pair $\left\{\hat{Q},\hat{D}\right\}$ at *μ*=0.5 generates the joint probabilities: $\pi_{11}=\pi_{44}=\frac{1}{4}\sin^{2}(\gamma /2)$ $\pi_{12}=\cos^{2}(\gamma /2)-\frac{1}{4}\sin^{2}(\gamma /2)(7-8\sin^{2}(\gamma /2))$, $\pi_{21}=\frac{1}{4}\sin^{2}(\gamma /2)(9-8\sin^{2}(\gamma /2))$. Thus, $p_{B}^{QD}=3-\frac{22}{4}\sin^{2}(\gamma /2)+4\sin^{4}(\gamma /2)$. It was $p_{B}^{QQ}=2-\frac{2}{4}\sin^{2}(\gamma /2)$ so that both pay-offs equalize as $1-5\sin^{2}(\gamma /2)+4\sin^{4}(\gamma /2)=0$, so that at $\gamma =2\arcsin(\frac{1}{2})=\pi /3$. Analogously, $p_{A}^{DQ}=-1+\frac{3}{4}\sin^{2}(\gamma /2)$ and $p_{A}^{QQ}=3-\frac{11}{4}\sin^{2}(\gamma /2)$ so that $p_{A}^{QQ}>p_{A}^{DQ}$, ∀*γ*. In consequence, the pair $\left\{\hat{Q},\hat{Q}\right\}$ at *μ*=0.5 is in NE for *γ*>γ*=*π*/3. As a reflection of this fact, in figure 7 from *γ*>*π*/3=1.047 both players resort again to the *Q* strategy (as shown in its figure 7*b*), and the form of both pay-offs emerging in the CA simulations is that shown in figure 2*a* under the *μ*=0.5 label, where the fairly locally linear functions intersect at *γ*=1.460 giving *p*_A_,*B*=1.777, as was demonstrated in the previous section.

Despite the structural proximity of both amplitude and phase-damping quantum noises mentioned at the end of §1 (only *K*_2_ varies), the pair (*Q*, *Q*) is not in NE with phase-damping noise for high values of *γ*. Consequently, in QSG-CA simulations with *μ*=0.5 phase-damping (not the shown here) the discontinuity observed with *μ*=0.5 amplitude-damping noise in figure 7 when *γ* approaches *π*/3 does not emerge in the pay-offs graph. The parameter graph shows in this scenario how the Samaritan player drifts to the *Q* strategy $({\bar{\theta }}_{A}\to 0$, ${\bar{\alpha }}_{A}\to \pi /2)$ when *γ* increases beyond *π*/3, but the beneficiary player does not accompany the Samaritan in this trend.

**Figure 8.**
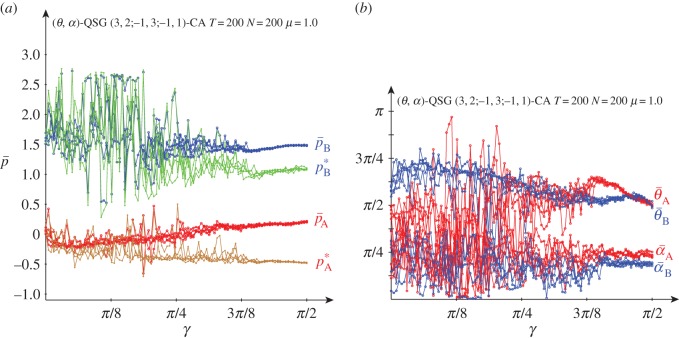
Five simulations at *T*=200 of a QSD-CA with variable entanglement *γ* and full noise. (*a*) Mean pay-offs and (*b*) mean quantum parameter values.

Figure 8 deals with the results obtained in the QSD-CA scenario of figure 7 but with full *μ*=1.0 noise. Full noise seems to impede the emergence of the (*Q*, *Q*) pair, so that the oscillations of both pay-offs not far from (−0.2, 1.5) shown in figures 3 and 7 for low *γ*, remain here for higher entanglement.

**Figure 9.**
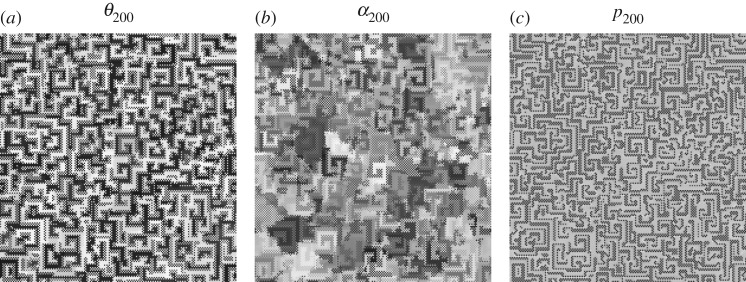
Quantum parameter (*θ*,*α*) and pay-off (*p*) patterns at *T*=200 in a simulation with *γ*=*π*/2 in the QSD-CA with full noise scenario of figure 8.

In figure 8, with high entanglement the (*θ*,*α*) parameters of both players tend to approach their middle levels (*π*/2,*π*/4). In this scenario, it is $\Pi =\frac{1}{4}\left(\begin{array}{cc} 1-\sin\gamma & 1\\ 1 & 1+\sin\gamma \end{array}\right)$, $p_{A}=\frac{1}{4}(1-3\sin\gamma )$, $p_{B}=\frac{1}{4}(6-2\sin\gamma )$. These pay-offs smoothly decrease as *γ* increases: *p*_A_ from 0.25 down to −0.5, *p*_B_ from 1.5 down to 1.0. The actual pay-offs $\left(\bar{p}\right)$ at *γ*=*π*/2 in figure 8*a* turn out to be over the expected (−0.5,1.0). This is due to spatial effects that make it difficult to estimate the actual mean pay-offs from the mean parameters. An example of spatial structure is given in figure 9, where the parameter values (and the pay-offs in consequence) show a kind of *maze*-like aspect. Figure 8*a* shows also the mean-field pay-offs (p*) achieved in a single hypothetical two-person game with players adopting the mean parameters appearing in the spatial dynamic simulation, those given in figure 8*b*. Namely,

U A∗=(eiα¯ A cos⁡θ¯ A2sin⁡θ¯ A2−sin⁡θ¯ A2e−iα¯ A cos⁡θ¯ A2)andU B∗=(eiα¯ B cos⁡θ¯ B2sin⁡θ¯ B2−sin⁡θ¯ B2e−iα¯ B cos⁡θ¯ B2).

In figure 8*a*, the mean-field pay-offs (p*_A_,p*_B_) are marked (brown,green), somehow following the (red,blue) colours of the actual mean pay-offs (${\bar{p}}_{A},{\bar{p}}_{B}$). As expected, the mean-field approaches fit fairly well the $p_{A}=\frac{1}{4}(1-3\sin\gamma )$, $p_{B}=\frac{1}{4}(6-2\sin\gamma )$ equations at high entanglement. Thus, at maximum *γ*=*π*/2, it is p*_A_≃−0.5, p*_B_≃1.0.

Although spatial effects arise in figures 3 and 7 before the emergence of the (*Q*, *Q*) pair, they are not particularly relevant, because the mean-field estimations do not appear in those figures. In the subsequent figures, the same criterion will be applied.

## 3. Unfair contests

Let us assume the unfair situation: a type of players is restricted to classical strategies $\tilde{U}(\theta ,0)$, whereas the other type of players may use quantum $\hat{U}(\theta ,\alpha )$ ones [27,28]. Since the dilemma in the SD is basically that of the charity player, the possibility of whether he can overcome the dilemma by restricting the beneficiary player to only classical strategies will be taken into account preferentially in this section, albeit the main results regarding the reversed unfair situation will be also reported.

**Figure 10.**
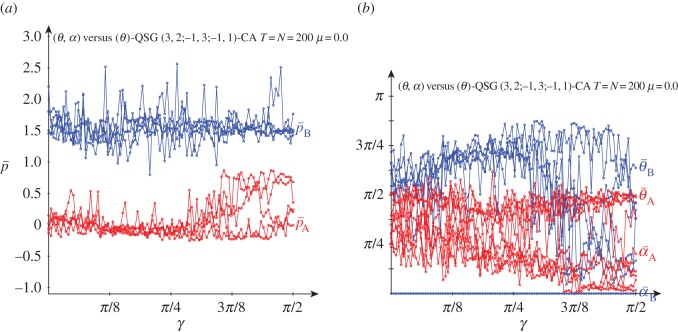
Five noiseless simulations at *T*=200 of a QSD-CA unfair quantum (*θ*,*α*)-player A (red) versus a classic *θ*-player B (blue) with variable entanglement *γ*. (*a*) Mean pay-offs $\bar{p}$ and (*b*) mean quantum parameter values.

Figure 10 deals with five noiseless simulations of a quantum (*θ*,*α*)-player A (red) versus a classic *θ*-player B (blue) in a QSD-CA with variable entanglement factor *γ*. The structure of the asymptotic mean pay-offs across the lattice ($\bar{p}$) of both players in this unfair scenario resembles that found in the fair scenario of figure 3 for not high *γ*, but extended now for all entanglement, without any discontinuity. Thus, rather unexpectedly, despite the fact that the beneficiary player B is restricted to classical strategies, he overrates the charity player A regardless of *γ* as ${\bar{p}}_{B}$ oscillates around 1.5, whereas ${\bar{p}}_{A}$ oscillates close to zero in most cases. Nevertheless, two simulations with high *γ* show higher values of ${\bar{p}}_{A}$, that may grow up to 0.5, somehow resolving the dilemma of player A in the weak sense making *θ*_A_=*π*/2 in the conventional (non-CA) game [7], or ${\bar{\theta }}_{A}≃\pi /2$ in CA simulations, as shown and in figure 10*b*.

In simulations in the scenario of figure 10 but with reversed unfairness: *θ*-player A versus (*θ*,*α*)-player B, the structure of the mean pay-offs versus *γ* (not shown here) resembles that shown in figure 10 in the (*θ*,*α*)-player A versus *θ*-player B scenario, although the quantum player B gets pay-offs slightly over 1.5, whereas the pay-offs of the classic player A stand slightly negative. The advantage of the quantum player (B) facing a classic player (A) is foreseeable; what may surprise here is that the quantum player B does not take a relevant advantage of his privileged role, getting pay-offs not far from those achieved in the opposite unfair scenario where he is the classic one.

### 3.1. Quantum noise

The simulations in the *α*_B_=0 unfair scenario with *μ*=0.5 noise (not shown here) produce roughly the same results as in the noiseless unfair scenario of figure 10, albeit the pay-offs of the charity player show a notably smaller variation around 1.5, and those of the beneficiary player become positive at a greater extent as in figure 10. Spatial effects are not relevant in figure 10, nor in the (*α*_B_=0, *μ*=0.5) simulations, so that the mean-field pay-offs estimations fit well to the actual ones in these unfair scenarios.

**Figure 11.**
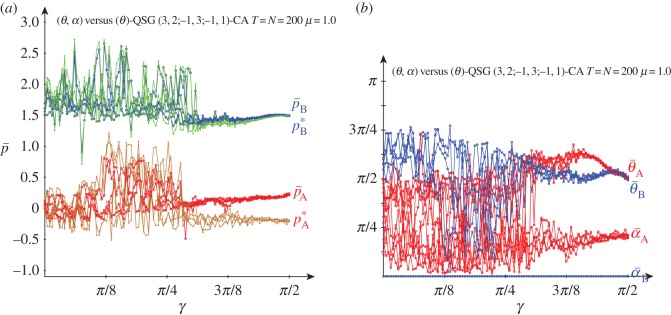
Five simulations at *T*=200 of a QSD-CA unfair quantum (*θ*,*α*)-player A (red) versus a classic *θ*-player B (blue) with variable entanglement *γ* and full noise. (*a*) Actual mean pay-offs $\bar{p}$ and mean-field pay-offs p*. (*b*) Mean quantum parameter values.

Figure 11 shows the results in the *α*_B_=0 unfair scenario of figure 10, but with *μ*=1.0 noise. The results regarding both the pay-offs and mean parameters shown in figure 11 are qualitatively similar to those achieved in the fair, *μ*=1.0 scenario of figure 8. Spatial effects arise with high entanglement, but oddly they seem to affect only the charity player A. Consequently, the parameter and the pay-offs spatial structures with high entanglement and full noise show a much fuzzified *maze*-like aspect compared to those in the fair scenario of figure 9.

**Figure 12.**
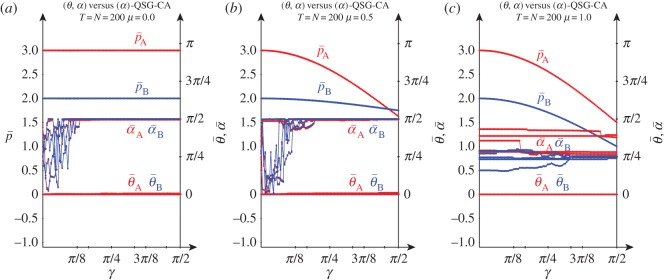
Five noiseless simulations at *T*=200 of a QSD-CA unfair quantum (*θ*,*α*)-player A (red) versus a (*α*)-player B (blue) with variable entanglement *γ*. (*a*) *μ*=0.0, (*b*) *μ*=0.5 and (*c*) *μ*=1.0.

Figure 12 deals with the case of an unfair game where the beneficiary player is allowed to resort only to the parameter *α* instead to *θ* as in the unfair simulations just before considered. In such a scenario, the (*Q*, *Q*) pair emerges in NE regardless of *γ* in the noiseless and *μ*=0.5 scenarios, as the beneficiary player can not resort to strategies such as D (or Loaf) which demands *θ*>0. As a result, in the noiseless figure 12*a* it is ${\bar{p}}_{A}=3$ and ${\bar{p}}_{B}=2$, and in the *μ*=0.5 noise (figure 12*b*), the $({\bar{p}}_{A}$, ${\bar{p}}_{B})$ pay-offs fit the expressions given in the quantum noise subsection of the introductory §1. In the full noise scenario of figure 12*c*, where ${\bar{\theta }}_{A}={\bar{\theta }}_{B}=0$, it is $\Pi =\left(\begin{array}{cc} \cos^{2}\frac{\gamma }{2} & 0\\ 0 & \sin^{2}\frac{\gamma }{2} \end{array}\right)$, so that p¯A=3 cos2⁡(γ/2), p¯B=2 cos2⁡(γ/2) regardless of the values of the *α* parameters. This is so because *θ*=0 makes $\hat{U}$ diagonal and it turns out that

$$({\hat{U}}_{A}⊗{\hat{U}}_{B})\rho_{1.0}^{*}({\hat{U}}_{A}⊗{\hat{U}}_{B})=\left(\begin{array}{cccc} e^{i(\alpha_{A}+\alpha_{B})}\;e^{-i(\alpha_{A}+\alpha_{B})} & 0 & 0 & 0\\ 0 & 0 & 0 & 0\\ 0 & 0 & 0 & 0\\ 0 & 0 & 0 & 0 \end{array}\right)=\left(\begin{array}{cccc} 1 & 0 & 0 & 0\\ 0 & 0 & 0 & 0\\ 0 & 0 & 0 & 0\\ 0 & 0 & 0 & 0 \end{array}\right).$$

## 4. Three-parameter strategies

This section deals with the full space of strategies SU(2), operating with three parameters (3P) as given in (4.1), so that a new *β* parameter is available.

4.1 U^(θ,α,β)=(eiα cos(θ2)eiβsin(θ2)−e−iβsin(θ2)e−iα cos(θ2)),θ∈[0,π]α,β∈[0,π2].

**Figure 13.**
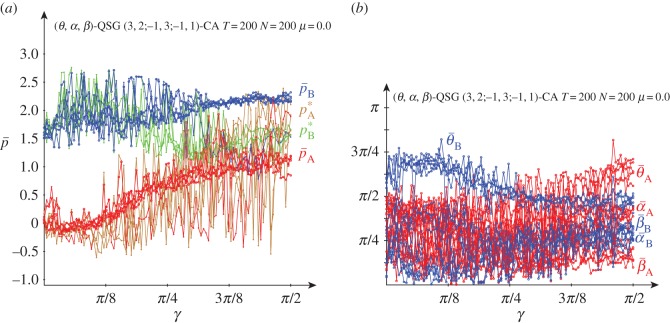
Five simulations at *T*=200 in a three-parameter QSD-CA with variable entanglement *γ* and no noise. (*a*) Actual mean pay-offs $\bar{p}$ and mean-field pay-offs p*. (*b*) Mean quantum parameter values.

Figure 13 deals with the results achieved in three-parameter strategies QSD-CA simulations free of noise. The actual mean pay-offs of both players monotonically increase their values as the entanglement increases, in the case of player A from approximately zero up approximately 1.5, in the case of player B from approximately 1.5 up to approximately 2.25. Thus, player B overrates player A all along the *γ* variation, albeit in a lower degree as *γ* grows. For low *γ*, the mean-field pay-off estimations fit fairly well the actual mean pay-offs, particularly in respect of player A, but as *γ* grows heavy spatial effects emerge, so that the actual mean pay-offs $\bar{p}$ become fairly stabilized, in contrast with the variable behaviour of p*, particularly in respect of player A. No particular structure becomes apparent in the parameter patterns in figure 13*b*, which corresponds to a rather erratic behaviour of the mean-field estimations.

### 4.1. Quantum noise

**Figure 14.**
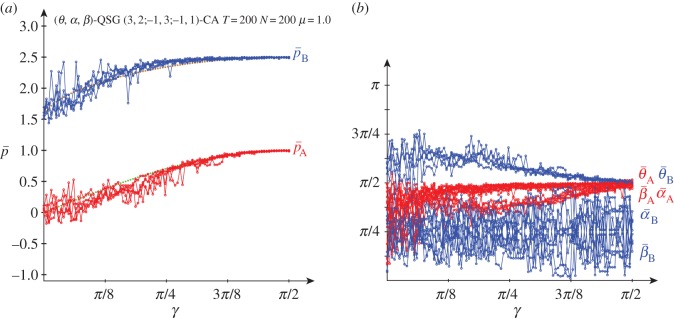
Five simulations at *T*=200 in a three-parameter QSD-CA with variable entanglement *γ* and *μ*=1.0 noise. (*a*) Actual mean pay-offs and (*b*) mean quantum parameter values.

In three-parameter strategies QSD-CA simulations with *μ*=0.5 noise (not shown here) roughly similar results as in the noiseless 3P scenario of figure 13 are achieved. Nevertheless, the form of the graphs of the actual mean and mean-field pay-offs are altered, much advancing the features observed in the 3P simulations with full noise shown below in figure 14. This is particularly true with respect to a notable decreasing in the erratic behaviour of the mean-field estimations, particularly those of player B.

Figure 14 deals with the results achieved in the scenario of figure 13, but with full noise. The actual mean pay-offs of both players monotonically increase their values as the entanglement increases, in the case of player A from approximately zero up approximately 1.5, in the case of player B from approximately 1.5 up approximately 2.0. Thus, player B overrates player A all along the *γ* variation, much as in figure 13, but with full noise in a more crisp manner. In fact, the actual mean pay-offs in figure 14 may be very well fitted by the equations: $p_{A}=\sin\gamma$, $p_{B}=\sqrt{\sin\gamma }$. At variance with what happens in figure 13, the mean-field pay-off estimations fit fairly well the actual mean pay-offs with full noise, so that such mean-field estimations have not been shown in figure 13. Again at variance with what happens in figure 13, some trends become apparent in the mean parameter graphics with full noise, as the mean parameter patterns of player A and ${\bar{\theta }}_{B}$ drift towards *π*/2.

**Figure 15.**
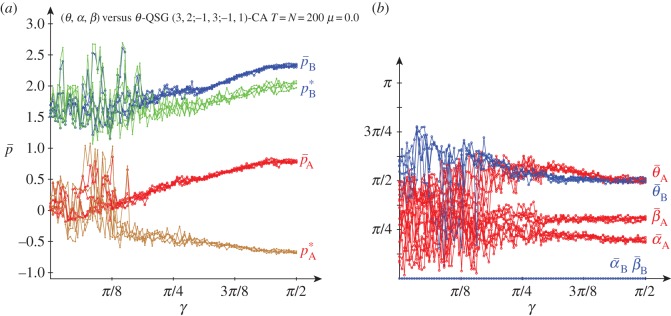
Five noiseless simulations at *T*=200 in the unfair three-parameter QSD-CA with entanglement *γ*. (*a*) Actual mean pay-offs $\bar{p}$ and mean-field pay-offs p*. (*b*) Mean parameter values.

The form of the graphs in the noiseless unfair three-parameter simulations shown in figure 15 apparently differ from that of its two-parameter counterpart in figure 10. Thus, as soon as the entanglement takes off, the mean parameters, and the mean pay-offs in consequence, become fairly stabilized: both $\bar{\theta }$ close to *π*/2, and ${\bar{\alpha }}_{A}$ and ${\bar{\beta }}_{A}$ close to *π*/4. Besides, high spatial effects emerge in figure 15 in contrast with their absence in figure 10. Spatial effects are particularly relevant regarding the charity player A, as the increase of the entanglement supports the increase of his actual mean pay-offs up to over 0.5, whereas his mean-field estimations decrease below −0.5. In simulations (not shown here) in the scenario of figure 15 but with reversed unfairness: *θ*-player A versus (*θ*,*α*,*β*)-player B, the classic charity player A gets negative pay-offs regardless of *γ*, but the pay-offs of the beneficiary player B keep not far from 1.5 for all *γ*, thus, surprisingly, getting smaller pay-offs than those achieved in the opposite unfair scenario of figure 15, where he is the classic one.

**Figure 16.**
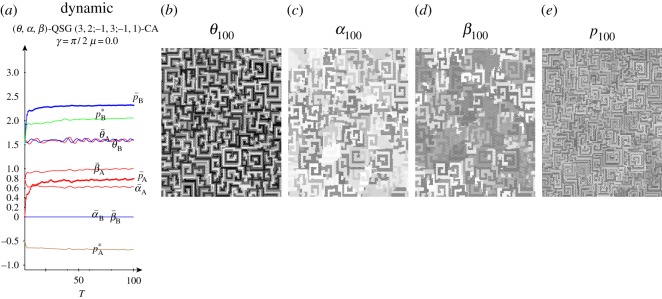
Dynamics up to *T*=100, and quantum parameter (*θ*,*α*) and pay-off patterns (*p*) at *T*=100 in a simulation with *γ*=*π*/2 in the QSD-CA unfair scenario of figure 15.

Figure 16 shows the dynamics up to *T*=100, together with the parameter and pay-off patterns at *T*=100 in a simulation with *γ*=*π*/2 in the QSD-CA unfair scenario of figure 15. Figure 16*a* indicates that both the quantum parameters and the pay-offs quickly reach their fairly permanent values, without a relevant initial transition time. As a result, the actual (and mean-field) values shown at *T*=200 in figure 15, do not significantly differ from those reached at *T*=100 (and even before) in figure 16. The parameter patterns values (and the pay-offs in consequence) in this figure show again, as in figure 9, a kind of *maze*-like aspect that is in the origin of the notable discrepancy between the actual and mean-field pay-offs, particularly that of the charity player A.

**Figure 17.**
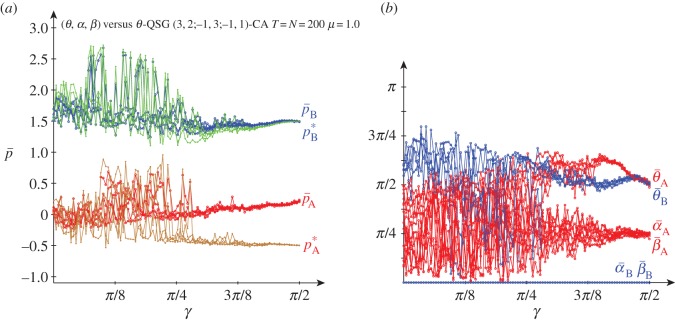
Five simulations at *T*=200 in the unfair three-parameter QSD-CA with entanglement *γ* and full noise. (*a*) Actual mean pay-offs $\bar{p}$, and mean-field pay-offs p*. (*b*) Mean quantum parameter values.

In three-parameter strategies QSD-CA unfair simulations with *μ*=0.5 noise (not shown here), although the general form of the graphs of the pay-offs and parameters versus *γ* is similar to that in the noiseless scenario of figure 13, the graphs are altered advancing the features observed in the 3P unfair simulations with full noise shown below in figure 17. In particular, with *μ*=0.5 noise the actual mean pay-offs of both players increase their values as *γ* increases at a lower degree compared to those achieved in the noiseless simulations.

In the *μ*=1.0 unfair 3P scenario of figure 17, where *α*_B_=*β*_B_=0, if *θ*_A_=*θ*_B_=*π*/2 and *α*_A_=*β*_A_=*π*/4 it is: $\Pi =\frac{1}{4}\left(\begin{array}{cc} 1-\sin\gamma & 1+\sin\gamma \\ 1-\sin\gamma & 1+\sin\gamma \end{array}\right)$, with associated pay-offs: *p*_B_=1.5, $p_{A}=0.25-1.5\sin\gamma$. The latter smoothly decreases from *p*_A_(*γ*=0)=0.25, down to *p*_A_(*γ*=*π*/2)=−0.50. Figure 17*b* indicates that the just described quantum parameters scenario applies with high entanglement in the simulations of this figure, and in consequence the mean–mean field pay-off estimations in figure 17*a* correspond with the theoretical values approximately from *γ*=*π*/4. Spatial effects minimally influence the actual mean pay-offs of the beneficiary player A in the CA simulations, whereas they support those of the charity player A so that he achieves no negative pay-offs.

In [7], two NE are reported in the noiseless scenario with full entangling when considering the initial density matrices *ρ*_*i*_=(|00〉〈00|+|11〉〈11|)/2 and *ρ*_*i*_=(|01〉〈01|+|10〉〈10|)/2. The strategies in NE in these scenarios have in common the parameters *θ*_A_=*θ*_B_=*π*/2 and *α*_B_+*β*_B_=*π*/2; with the first density matrix it is *α*_A_+*β*_A_=*π* and with the second one *α*_B_+*β*_B_=0. The results of their corresponding CA simulations are not shown here, but they are able to detect such NE in a straightforward manner, i.e. free of spatial effects, reporting in both cases non-negative pay-offs to the Samaritan player. In the particular case of full entangling, it is ${\bar{p}}_{A}=1.0$, ${\bar{p}}_{B}=2.5$ in both scenarios. Thus, the general 3P-SU(2) operators may become a powerful tool when the players share a classically correlated state.

## 5. Conclusion

A spatial formulation of the iterated QSD game with arbitrary entangling is studied in this work. The game is played in the cellular automata (CA) manner, i.e. with local and synchronous players’ interaction. The evolution is achieved via imitation of the best-paid neighbour of the two players confronted in the SD: the charity and the beneficiary player. The paper considers both the general case of players accessing the full space of quantum strategies, which allows for three parameters (3P), and the particular case of strategies restricted to a subset of them allowing for only two parameters (2P). Although the restriction to two parameters may be criticized, the 2P model is a good and widely used test-bed to show how the quantum approach in game theory may *solve* dilemmas, by allowing for NE strategies out of the scope of the classic approach as summarized below.

In fair contests (facing two quantum players), the CA simulations allow for the emergence of the NE strategies. Thus, with low entanglement the strategy in NE in the classic game dominates the scene preserving the structural SD trend favouring the beneficiary player. But with high entanglement, the so-called *Q* strategy is adopted by both players in a dramatically defined manner, somehow *resolving* the dilemma as mutual *Q* provides the pay-offs of the (charity-Aid, beneficiary-Work) choice.

With quantum noise, the dynamics demands higher entanglement for the emergence of the (*Q*,*Q*) pair. In our study (*Q*,*Q*) emerges from *γ*>*π*/4 in noiseless simulations, and from *γ*>*π*/3 with amplitude damping quantum noise at middle level. In the extreme case, full noise impedes the emergence of the (*Q*,*Q*) pair. Besides, noise notably alters the pay-offs of mutual *Q*.

In the unfair quantum versus classic scenario, the beneficiary player over-scores the charity player regardless of the degree of entanglement and the degree of noise. This is so even if the beneficiary player is the classic one, albeit in this case the charity player may achieve relatively high positive pay-offs in noiseless simulations with high entangling.

Mean-field approaches of the actual mean pay-offs fail as a tool for estimation due to spatial effects in unfair contests and in simulations with high noise.

General pay-off matrices [7] and other noise types [16,29–31] are to come under scrutiny in subsequent studies on iterated quantum games in the spatial context. Other quantization schemes [32–35], multi-party games [36,37] as well as games with imperfect information [23,38–41] deserve particular studies.

Deviations from the canonical cellular automata paradigm adopted here may lead to more realistic models. Particularly, structurally dynamic CA, asynchronous updating, spatial dismantling and, last but not least, dynamics with embedded tuneable memory of past pay-offs and parameter values [42].

## Footnotes

1 In the five simulations of figure 3 at *γ*=0 it is: $\left({\bar{\theta }}_{A},{\bar{\theta }}_{B})=(1.568,1.710\right)$, (0.741, 2.068), (1.239, 2.256), (1.240, 1.906), (1.606, 2.086), with associated mean-field pay-offs: (*p**_A_,*p**_B_)=(0.147,1.503), (0.006, 2.414), (−0.238, 1.928), (0.113, 1.878), (−0.125, 1.456), very close to the actual pay-offs: $({\bar{p}}_{A},{\bar{p}}_{B}$)=(0.149, 1.502), (−0.054, 2.201), (−0.130, 1.813), (0.027, 1.798), (−0.087, 1.456), as shown in figure 3*a*.

## Data accessibility

The data supporting the graphs in the figures of this article are available from the Dryad Digital Repository: http://dx.doi.org/10.5061/dryad.km170 [43].
